# Molecular crosstalk between MASLD and IVDD revealed through integrated biomarker discovery analysis

**DOI:** 10.3389/fimmu.2026.1703972

**Published:** 2026-01-26

**Authors:** Guohao Wang, Yongming Liu, Xingchao Shen

**Affiliations:** 1 Department of traumatic orthopedics, Shaoxing Traditional Chinese Medicine Hospital Affiliated to Zhejiang University of Chinese Medicine, Shaoxing, China; 2 Department of Rehabilitation Medicine, Shanghai Tenth People’s Hospital, Tongji University School of Medicine, Shanghai, China; 3 Department of Spine Surgery, Shaoxing Traditional Chinese Medicine Hospital Affiliated to Zhejiang University of Chinese Medicine, Shaoxing, China

**Keywords:** biomarkers, chronic inflammation, IVDD, MASLD, molecular crosstalk, WGCNA

## Abstract

**Background:**

Non-alcoholic fatty liver disease (NAFLD), recently retermed as metabolic dysfunction-associated steatotic liver disease (MASLD), and intervertebral disc degeneration (IVDD) are major health burdens with rising prevalence. Despite affecting different organ systems, emerging evidence suggests potential molecular crosstalk between these conditions. However, the underlying mechanisms remain poorly understood.

**Methods:**

We employed comprehensive bioinformatics analysis to investigate shared pathogenic mechanisms between MASLD and IVDD through integrated bulk and single-cell RNA sequencing datasets. Weighted gene co-expression network analysis and LASSO regression were used to identify common biomarkers. Experimental validation was performed using blood samples from patients and controls.

**Results:**

Transcriptomic profiling revealed distinct molecular signatures: MASLD showed enrichment in metabolic pathways (cholesterol metabolism, PPARγ signaling), while IVDD exhibited cellular signaling activation (MAPK, PI3K-AKT pathways). Four shared biomarkers were identified through LASSO regression: STAB2, RAPGEFL1, IGF1, and ZNF285. Experimental validation confirmed significant STAB2 upregulation and IGF1 downregulation in both diseases, with enhanced alterations in concurrent MASLD-IVDD patients. Through single-cell analysis of 10,388 NAFLD cells and 35,846 IVDD cells, Scissor analysis was employed to identify disease-associated cell populations and revealed two additional common biomarkers (PHACTR1 and RIPOR2), with experimental validation demonstrating significant alterations in patients with concurrent MASLD-IVDD. Furthermore, immune communication analysis identified GALECTIN as the predominant shared signaling pathway.

**Conclusions:**

This study provides preliminary evidence for molecular crosstalk between MASLD and IVDD, suggesting systemic metabolic dysfunction may influence distant tissue pathology through shared inflammatory and metabolic pathways.

## Introduction

Non-alcoholic fatty liver disease (NAFLD), recently retermed as metabolic dysfunction-associated steatotic liver disease (MASLD), has emerged as the most prevalent chronic liver disease globally, affecting approximately 25% of the world’s population and representing a significant economic burden on healthcare systems worldwide (1, 2). The pathophysiology of MASLD involves complex metabolic dysregulation, including disrupted lipid metabolism, altered glucose homeostasis, and impaired insulin sensitivity, which collectively contribute to systemic metabolic dysfunction (3, 4). This systemic metabolic dysfunction affects not only the liver itself but may also influence the metabolic environment of distant tissues through multiple mechanisms (5). Patients with MASLD commonly exhibit increased oxidative stress, characterized by enhanced reactive oxygen species production and compromised antioxidant defense systems, which can affect various organs throughout the body via the circulatory system (6). MASLD progression is associated with chronic low-grade systemic inflammation, characterized by hepatic production and release of pro-inflammatory cytokines (tumor necrosis factor-α, interleukin-6, interleukin-1β), chemokines, and acute-phase proteins into the systemic circulation (7). This creates a persistent inflammatory milieu that extends beyond the liver and can influence homeostasis in distant tissues. For intervertebral discs, which normally exist in a relatively immune-privileged environment, exposure to systemic inflammatory mediators can activate catabolic signaling pathways, promote matrix metalloproteinase expression, induce cellular senescence, and shift the balance from extracellular matrix synthesis to degradation—hallmarks of disc degeneration (8–10). Furthermore, MASLD-associated insulin resistance and glucose metabolic abnormalities alter systemic nutrient utilization patterns, potentially affecting glucose-dependent tissues such as intervertebral discs, where cells rely on efficient glucose metabolism for extracellular matrix synthesis and cellular homeostasis (11).

Similarly, the metabolic environment plays a crucial role in intervertebral disc degeneration (IVDD). IVDD represents a common clinical manifestation of spinal degeneration and serves as a major contributor to low back pain, constituting a prevalent age-related condition (12, 13). Approximately 60–80% of the world’s population is affected by IVDD, resulting in a substantial social and economic burden (14). The etiology of IVDD involves multifactorial mechanisms including aging, mechanical stress, genetic predisposition, and environmental factors (15). IVDD is characterized by progressive breakdown of the intervertebral disc structure, involving complex cellular processes including extracellular matrix degradation, cellular senescence, and altered cellular signaling (16–18). However, metabolic dysfunction manifested by metabolic reprogramming toward glycolysis, mitochondrial dysfunction, and altered cellular energy production promotes IVDD disease progression (19). This metabolic dysfunction further results in lactate accumulation, tissue acidosis, and impairment of extracellular matrix integrity (20). Additionally, reactive oxygen species produced by dysfunctional mitochondria induce oxidative stress that damages cellular components and activates pathways leading to cellular senescence (21). These metabolic characteristics of IVDD suggest that systemic metabolic disorders could significantly impact disc health. Given that disc cells rely heavily on glucose metabolism and are sensitive to changes in nutrient availability, pH balance, and oxidative stress, conditions that affect systemic metabolism, such as diabetes, obesity, and metabolic syndrome, may indirectly contribute to disc degeneration. The hypoxia-responsive signaling pathways essential for maintaining metabolic homeostasis in disc cells further underscore the potential vulnerability of the intervertebral disc to systemic metabolic dysfunction (22). Understanding these metabolic connections provides a theoretical framework for investigating potential molecular crosstalk between systemic metabolic diseases and intervertebral disc degeneration.

Epidemiological evidence suggests potential associations between MASLD and IVDD, indicating that despite affecting different organ systems, these conditions may share common underlying mechanisms. Population-based cohort studies have demonstrated that metabolic syndrome components, including overweight, hypertension, and impaired glucose tolerance, are significantly associated with intervertebral disc degeneration across cervical, thoracic, and lumbar regions, with overweight showing particularly strong associations (23). Furthermore, Mendelian randomization studies have provided evidence that metabolic disturbances, including type 2 diabetes and elevated triglycerides, have more significant causal effects on IVDD than biomechanical alterations, suggesting that systemic metabolic dysfunction may be a primary driving factor rather than a secondary consequence (24). Additionally, case-control studies have found that dyslipidemia may serve as a useful predictor of IVDD (25). These epidemiological observations align with mechanistic understanding that MASLD-driven metabolic perturbations—including dyslipidemia, insulin resistance, and systemic inflammation—create a pathogenic microenvironment that could accelerate degenerative processes in metabolically vulnerable tissues like intervertebral discs. Despite these observed epidemiological associations and potential mechanistic connections, systematic investigation of shared molecular signatures between MASLD and IVDD remains limited.

Therefore, MASLD and IVDD share three interconnected pathological processes that provide a conceptual framework for understanding their potential crosstalk (1): Metabolic dysfunction: Both conditions feature disrupted cellular energy metabolism, with MASLD characterized by aberrant lipid accumulation and altered glucose handling in hepatocytes, while IVDD involves metabolic reprogramming toward glycolysis, mitochondrial dysfunction, and impaired nutrient sensing in disc cells (2). Chronic inflammation: MASLD progression is associated with activation of inflammatory signaling cascades and release of pro-inflammatory mediators that can propagate systemically, while IVDD pathogenesis involves inflammatory cytokine-mediated cellular damage and tissue catabolism (3). Extracellular matrix breakdown: Both diseases feature imbalanced matrix homeostasis, with excessive matrix metalloproteinase activity and reduced synthesis of structural proteins—hepatic fibrosis in MASLD and proteoglycan/collagen degradation in IVDD. We hypothesize that these shared pathological processes may be coordinately regulated through common molecular mechanisms, and that liver-derived metabolic and inflammatory factors could directly influence disc tissue homeostasis, creating a pathogenic axis linking these seemingly disparate conditions.

**Figure 1:**
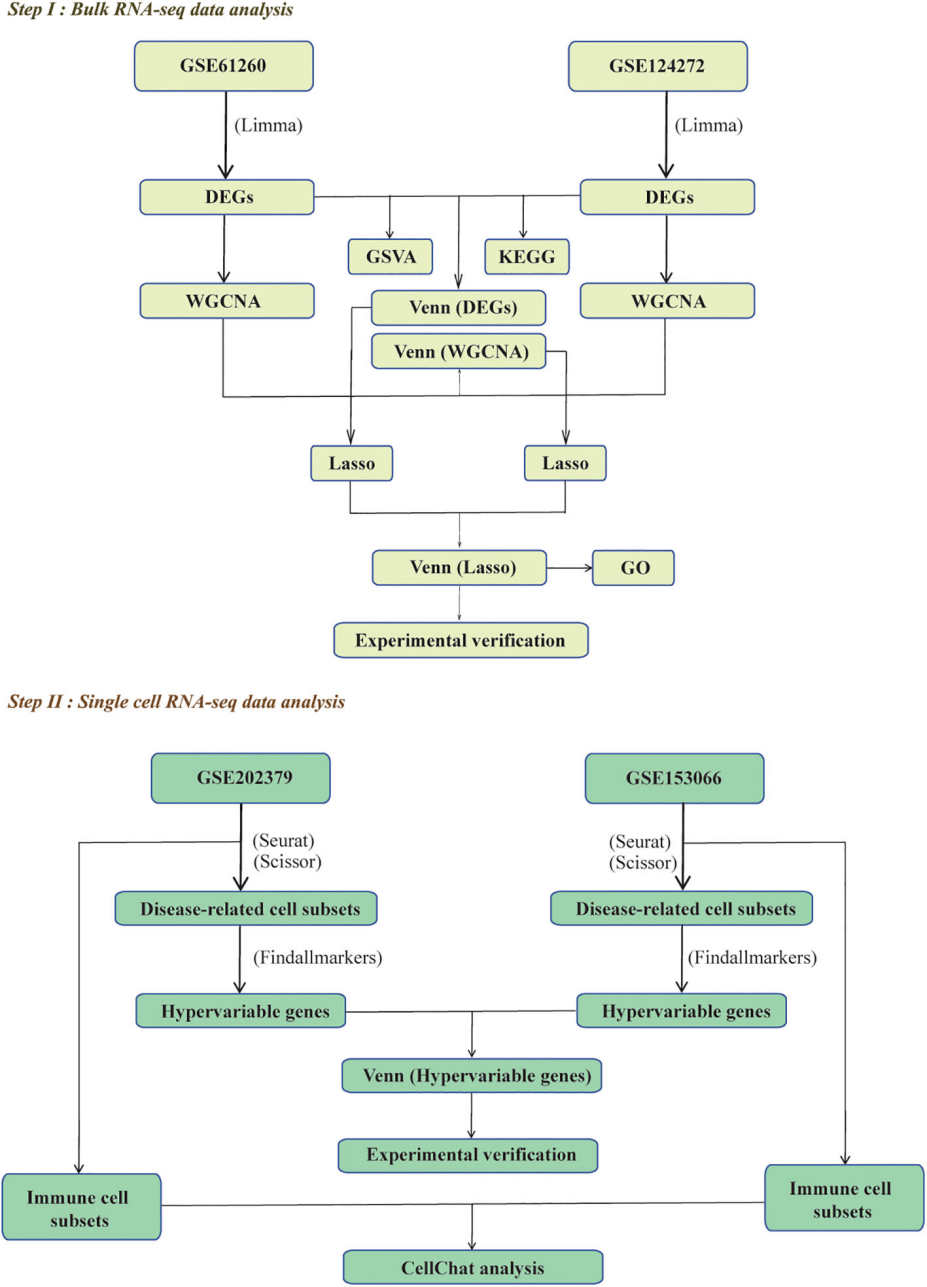
The scheme of research.

Current understanding of MASLD-IVDD associations relies primarily on epidemiological observations, while the underlying molecular mechanisms linking these diseases remain poorly characterized. Bulk RNA sequencing provides insights into tissue-level transcriptomic changes but cannot resolve cellular heterogeneity or identify disease-associated cell populations. Conversely, single-cell RNA sequencing captures cellular diversity but may miss coordinated gene expression patterns observable at the tissue level. Integration of both approaches is therefore essential to achieve comprehensive understanding of disease mechanisms. This study addresses these critical knowledge gaps by employing comprehensive bioinformatics analysis to systematically investigate molecular crosstalk between MASLD and IVDD. By integrating bulk RNA sequencing and single-cell RNA sequencing datasets from both conditions, we seek to identify common differentially expressed genes, biomarkers, and cellular communication patterns that may underlie the observed epidemiological associations. Through differential gene expression analysis, weighted gene co-expression network analysis, machine learning-based feature selection, and single-cell analysis, combined with quantitative PCR validation of candidate biomarkers in human blood samples, we aim to provide a comprehensive understanding of the molecular crosstalk between these two conditions. The identification of shared biomarkers and molecular pathways between MASLD and IVDD could have significant implications for understanding systemic metabolic disease mechanisms and developing integrated diagnostic and therapeutic approaches (**Figure 1**).

## Materials and methods

### Dataset collection and preprocessing

This study analyzed four RNA sequencing datasets from the GEO NCBI database to investigate the molecular mechanisms underlying MASLD and IVDD. For single-cell analysis, we utilized GSE202379 (MASLD) and GSE153066 (IVDD) datasets. Additionally, bulk RNA-seq datasets GSE61260 (MASLD) and GSE124272 (IVDD) were collected for comprehensive transcriptomic profiling and biomarker discovery. All datasets underwent quality assessment and normalization procedures to ensure compatibility for downstream integrative analysis.

### Differential gene expression analysis

To identify genes significantly associated with disease phenotypes, differential expression analysis was performed using the limma package (v3.56.2) in R. Linear models with empirical Bayes moderation were employed to assess gene-level changes while accounting for sample variability (26). Differentially expressed genes (DEGs) were defined using stringent criteria: |log2FC| > 0.5 and FDR-adjusted p-value < 0.05. Volcano plots were generated to visualize the distribution of up-regulated and down-regulated genes. Gene Set Variation Analysis (GSVA) was subsequently performed to validate the distribution of identified DEGs across different sample groups and datasets, ensuring the robustness of our findings.

### Weighted gene co-expression network analysis

WGCNA was performed using bulk RNA-seq data to identify gene modules associated with MASLD and IVDD progression. Hierarchical clustering analysis was conducted using the average linkage method based on topological overlap matrix (TOM) dissimilarity (27). To establish a scale-free network topology, soft-thresholding power values ranging from 1 to 30 were tested, with the optimal power value (β = 10) selected based on scale-free topology fit (R² > 0.8) while maintaining sufficient gene connectivity (28). The DynamicTreeCut algorithm was employed for initial module detection, followed by module merging based on eigengene similarity (threshold = 0.25). Gene significance (GS) and module membership (MM) were calculated for trait-module correlation analysis. Modules with |correlation coefficient| ≥ 0.3 and p-value < 0.05 were considered significantly associated with disease traits. Cross-validation between DEGs identified by limma and WGCNA module genes was performed to obtain high-confidence candidate genes.

### Biomarker selection using LASSO regression

To identify the most predictive biomarkers and control model overfitting, Least Absolute Shrinkage and Selection Operator (LASSO) regression was performed using the glmnet package (v4.1-8) in R. LASSO regression was specifically chosen for its ability to perform automatic variable selection and reduce model complexity through L1 regularization, making it particularly suitable for high-dimensional genomic data with potential multicollinearity. Input data consisted of normalized gene expression profiles from disease-associated samples identified through the intersection of DEGs and WGCNA module genes, ensuring that only biologically relevant candidates were considered. Clinical phenotypes (disease vs. control) served as response variables in the binomial regression model. The optimal regularization parameter (λ) was determined through 10-fold cross-validation based on binomial deviance minimization, with two critical λ values considered: λ.min (minimum cross-validated error) and λ.1se (most regularized model within one standard error of minimum) (29). We selected λ.1se (the most regularized model within one standard error of the minimum cross-validated error) to prioritize model parsimony and reduce overfitting risk, with final biomarker selection based on four stringent criteria (1): non-zero coefficients at the optimal λ.1se value (2), consistent selection across multiple cross-validation runs with ≥80% selection frequency (3), established biological relevance based on literature review and functional annotation, and (4) detectable expression in peripheral blood to ensure clinical applicability for non-invasive biomarker development.

### Single-cell RNA sequencing data processing and analysis

Single-cell RNA-seq data analysis was performed using the Seurat software package (version 5.1.0). Quality control criteria included (1): genes expressed in ≥3 cells (2), cells expressing ≥200 genes (3), unique molecular identifiers (UMIs) ≥600 (4), mitochondrial gene percentage <10%, and (5) hemoglobin gene percentage <1%. After quality filtering, 10,388 MASLD cells and 35,846 IVDD cells were retained for downstream analysis. Data normalization, scaling, and cell cycle effect removal were performed using the “SCTransform” function (30). The top 3,000 highly variable genes were identified for dimensionality reduction. Principal Component Analysis (PCA) was conducted, and the Harmony algorithm was applied to correct batch effects. The first 30 principal components were selected for integration. Uniform Manifold Approximation and Projection (UMAP) was employed for non-linear dimensionality reduction and visualization (31). Cell clustering was performed using the Louvain algorithm, resulting in 14 clusters for MASLD and 19 clusters for IVDD datasets.

To ensure data quality and minimize technical artifacts, we implemented comprehensive batch effect correction procedures. The Harmony algorithm was applied to integrate cells from multiple biological samples while preserving genuine biological variation. Integration quality was assessed through multiple criteria (1): visual inspection of UMAP projections before and after integration to confirm sample mixing (2), calculation of entropy of batch mixing scores to quantitatively evaluate integration success (all cell types achieved mixing entropy >0.75, indicating effective batch correction), and (3) verification that post-integration clustering was driven by canonical cell type markers rather than sample origin. During downstream differential expression analysis, sample identity was included as a covariate in statistical models to account for any residual sample-specific effects. The concordance of single-cell findings with independent bulk RNA-seq datasets further validated the robustness of our results against potential batch-related biases.

### Cell type annotation and marker gene identification

Cell type annotation was performed based on canonical marker genes from literature and the CellMarker2.0 database. The “FindAllMarkers” function in Seurat was used to identify differentially expressed genes for each cluster (LogFC threshold = 0.25, min.pct = 0.1) (32). Manual annotation was conducted by comparing cluster-specific markers with established cell type signatures. MASLD tissues were annotated into 9 distinct cell types, while IVDD tissues were classified into 8 cell types. Feature plots were generated to visualize the expression patterns of key marker genes across different cell populations.

### Disease-associated cell identification using scissor analysis

The Scissor (v2.0.0) software package was employed to integrate single-cell and bulk RNA-seq datasets for identifying disease-associated cell populations. Binary phenotype classification was performed with disease samples assigned a phenotypic index of 1 and healthy controls assigned 0. Scissor+ cells were defined as positively correlated with disease phenotype, while Scissor- cells were negatively correlated. Hypergeometric distribution tests were used to evaluate the statistical significance of cell subset enrichment within respective phenotypes. Results were visualized using chord plots to demonstrate the relationship between cell subsets and their associated phenotypes. The “FindAllMarkers” function was applied to identify highly variable genes within disease-associated cell subsets, followed by Venn diagram analysis to determine common markers between MASLD and IVDD.

### Intercellular communication analysis

Immune cells were extracted from both MASLD and IVDD single-cell datasets for intercellular communication analysis using the CellChat package (V1.6.1). The analysis focused on ligand-receptor interactions within the immune microenvironment of both diseases. Communication networks were visualized to identify key signaling pathways, dominant cell types in intercellular communication, and specific ligand-receptor pairs. Enrichment bubble plots were generated to highlight the most significant receptor-ligand interactions. Common signaling pathways between MASLD and IVDD immune microenvironments were identified through comparative analysis.

### Functional enrichment analysis and pathway annotation

Gene Ontology (GO) and Kyoto Encyclopedia of Genes and Genomes (KEGG) enrichment analyses were performed using the Metascape platform with parameters set to “Homo sapiens” and significance threshold of p < 0.05. Biological processes (BP), cellular components (CC), and molecular functions (MF) were analyzed for identified biomarkers (33). Network visualization was conducted to demonstrate functional associations between biomarkers and their enriched biological processes. Clustering analysis was performed to group biomarkers based on their functional similarities and expression patterns across disease samples.

### Experimental validation

Blood specimens were obtained from Shaoxing Hospital of Traditional Chinese Medicine under an institutional ethics committee-approved protocol for human sample collection: No. 010-01 (Research, 2023, Audited by Ethics Committee) of Shaoxing Traditional Chinese Medicine Hospital, with whole blood samples containing ethylenediaminetetraacetic acid (EDTA) collected from healthy controls (n=5), MASLD patients (n=5), IVDD patients (n=5), and MASLD -IVDD patients (n=5). Total RNA was isolated using the Blood RNA Kit (TransGen Biotech) following manufacturer’s instructions, and complementary DNA was subsequently synthesized using the cDNA Synthesis Kit (TransGen Biotech). Gene expression analysis was performed using SYBR Green-based quantitative PCR premix (TransGen Biotech) on an Applied Biosystems QuantStudio 6 Real-Time PCR System, with each 20 µL reaction mixture containing 2×PerfectStart Green qPCR SuperMix (10 µL), forward and reverse primers (0.4 µL each, 10 µM), cDNA template (2 µL), and nuclease-free water (7.2 µL). All reactions were conducted in triplicate with β-actin as the internal reference gene, and relative gene expression levels were calculated using the comparative Ct method (2^-ΔΔCt^).

### Statistical analysis

Statistical analyses were conducted using R software (v4.3.0). The hypergeometric distribution test was employed for cell subset enrichment analysis. The Wilcoxon rank-sum test was used for continuous variable comparisons between groups. Multiple testing correction was applied using the Benjamini-Hochberg method where appropriate. Statistical significance was defined as p < 0.05 with 95% confidence intervals (CI). Data visualization was performed using ggplot2 and other relevant R packages. Sample clustering and heatmap generation were conducted to validate biomarker expression patterns across different disease states.

## Results

### Transcriptomic profiling reveals distinct gene expression signatures in MASLD and IVDD progression

**Figure 2:**
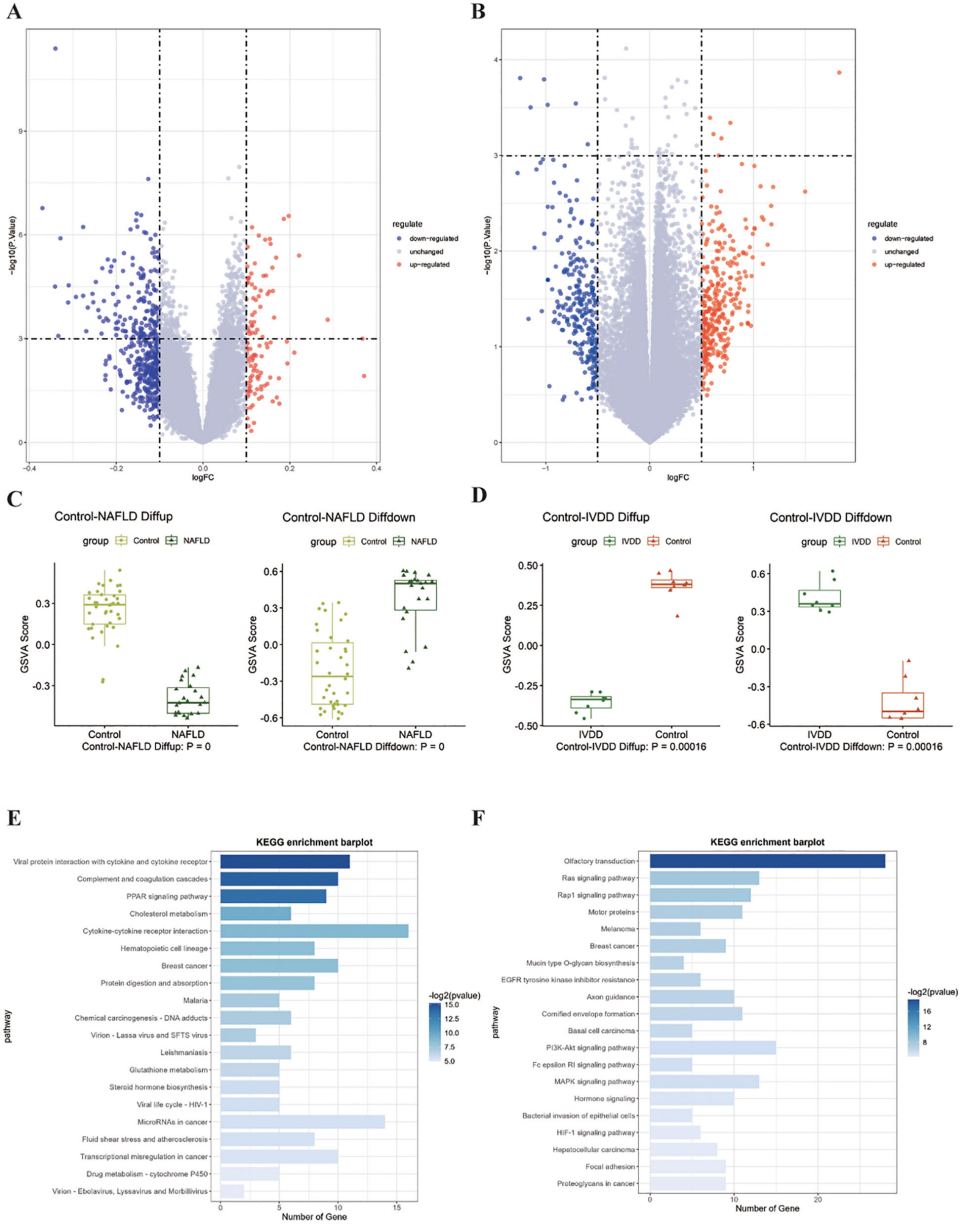
Comprehensive transcriptomic analysis identifies disease-specific molecular signatures. **(A)** Volcano plots of MASLD. **(B)** Volcano plots of MASLD. **(C)** GSVA analysis of MASLD. **(D)** GSVA analysis of IVDD. **(E)** KEGG analysis of MASLD. **(F)** KEGG analysis of IVDD.

Comprehensive differential gene expression analysis of bulk RNA-seq datasets unveiled distinctive transcriptional landscapes associated with disease pathogenesis. Volcano plot visualization demonstrated significant alterations in gene expression profiles (**Figures 2A, B**). To validate the reliability and distribution patterns of identified DEGs, GSVA was employed across different datasets and sample groups. The GSVA results confirmed consistent enrichment patterns of disease-associated gene signatures, demonstrating the robustness of our transcriptomic findings and supporting the biological relevance of identified molecular alterations (**Figures 2C, D**). The KEGG analysis revealed substantial transcriptomic perturbations with distinct pathway signatures in both conditions. As shown in **Figure 2E**, MASLD demonstrated pronounced enrichment in metabolic regulatory pathways, including cholesterol metabolism, glutathione metabolism, and protein absorption processes, alongside steroid hormone biosynthesis, PPARγ signaling cascades, and cytokine-receptor interaction networks. In contrast, IVDD exhibited significant activation of cellular signaling pathways, predominantly featuring Rap1 signaling, RAS signaling, MAPK cascades, PI3K-AKT pathway, and HIF-1 signaling networks (**Figure 2F**).

### Network-based gene module discovery through weighted co-expression analysis

**Figure 3:**
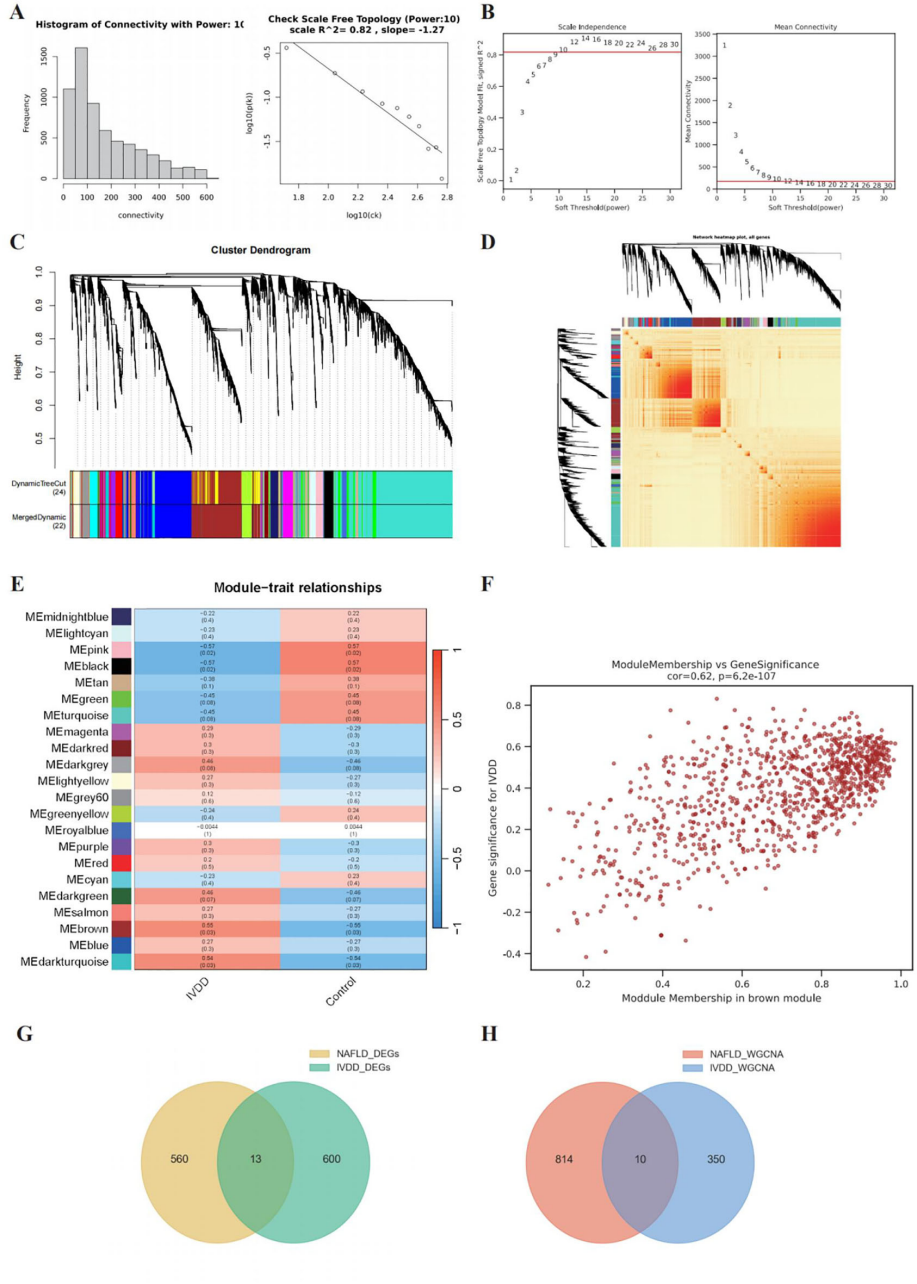
Systematic gene co-expression network analysis reveals disease-associated molecular modules. **(A, B)** Hierarchical clustering dendrograms constructed using topological overlap matrix dissimilarity with average linkage method. **(C)** Initial clustering using DynamicTreeCut algorithm identifying 24 preliminary modules. **(D)** Final merged clustering resulting in 22 biologically meaningful co-expression modules after dynamic branch cutting. **(E, F)** Detailed visualization of selected dendrogram branches highlighting distinct module separation (colored branches), module-specific expression patterns, and functional annotations for representative modules. **(G, H)** Venn diagram analysis showing intersection of DEGs identified by limma algorithm and WGCNA module genes, yielding cross-validated gene candidates.

To elucidate the underlying molecular networks driving disease progression, we implemented WGCNA using hierarchical clustering based on topological overlap matrix dissimilarity and average linkage methodology. Optimal soft-thresholding power selection was achieved through systematic evaluation of correlation coefficients and network connectivity parameters across a range from 1 to 30, ultimately determining power = 10 as the optimal value for achieving scale-free network topology while maintaining adequate gene connectivity (**Figures 3A, B**). The comprehensive network construction process successfully partitioned 6,362 genes into 22 distinct co-expression modules, with the gray module representing unassigned genes excluded from further analysis. Through correlation heatmap analysis and trait-module association assessment, we identified the brown module as exhibiting the strongest correlation with disease phenotypes (|correlation coefficient| ≥ 0.3, p < 0.05) (**Figures 3C–E**). Detailed examination of gene significance (GS) and module membership (MM) relationships within selected modules revealed high correlation coefficients between individual gene expression levels and both disease traits and module eigengenes, validating the biological coherence of identified modules (**Figure 3F**). Strategic intersection analysis between WGCNA-derived module genes and limma-identified DEGs yielded high-confidence cross-validated gene sets for subsequent biomarker discovery (**Figures 3G, H**).

### Machine learning-based biomarker discovery through LASSO regression analysis

**Figure 4:**
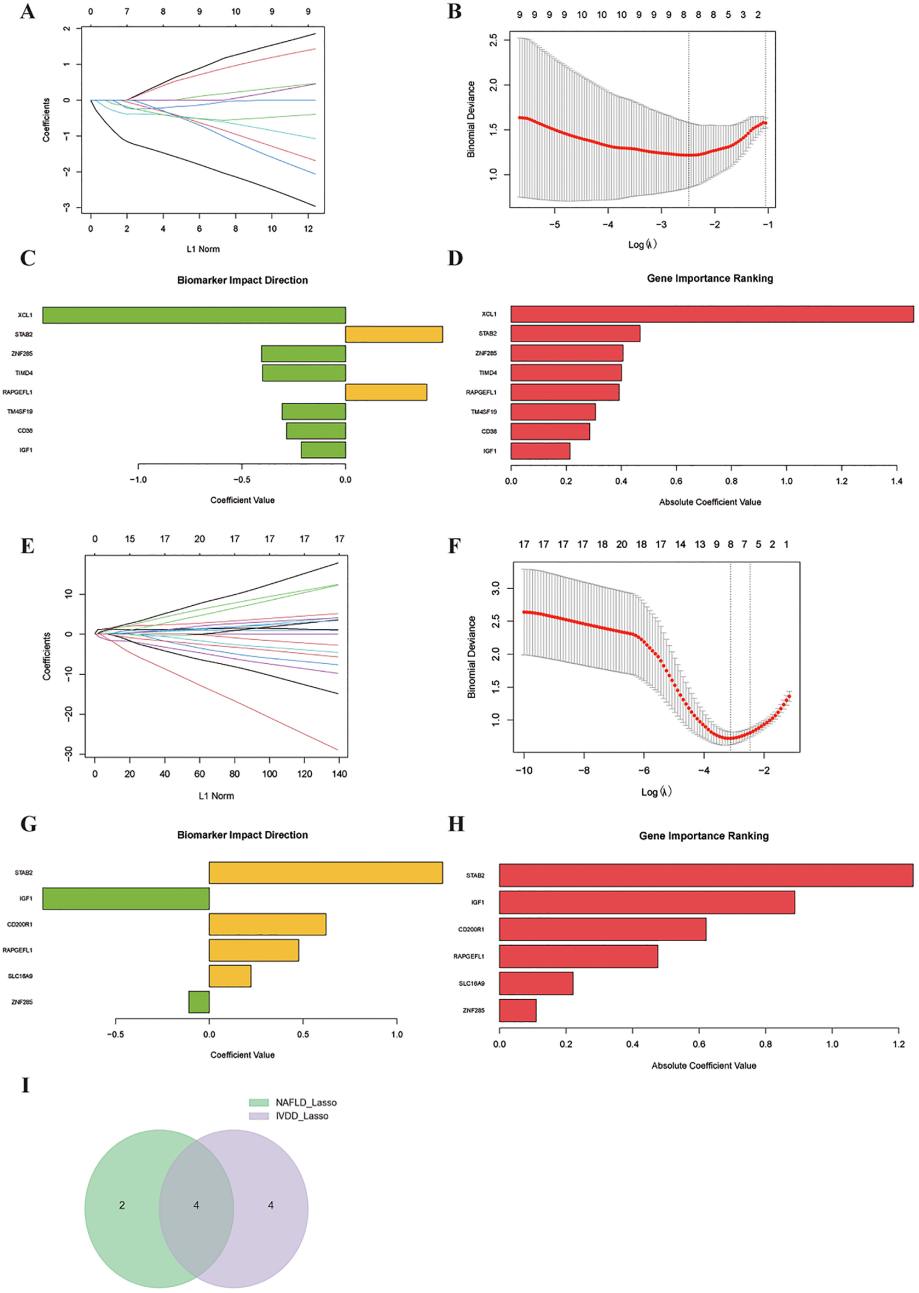
Machine learning-driven biomarker identification and validation through LASSO regression. **(A, B)** MASLD biomarker discovery: Left panel shows coefficient paths during variable selection with L1 norm (x-axis) and coefficient values (y-axis). Numbers indicate non-zero coefficients at critical λ values. Right panel displays binomial deviance versus log(λ) with optimal λ selection (dashed vertical lines). **(C, D)** Extended biomarker analysis for MASLD showing consistent feature selection patterns through corresponding coefficient paths and deviance plots. **(E, F)** IVDD biomarker identification: Coefficient paths and optimal λ selection following identical analytical framework. **(G, H)** Comprehensive biomarker analysis of IVDD. **(I)** Venn diagram intersection analysis identifying four shared biomarkers between NAFLD and IVDD.

Our systematic LASSO regression approach successfully identified disease-specific biomarker signatures while effectively controlling model complexity and overfitting. The coefficient regularization path demonstrated progressive variable selection as the penalty parameter (λ) increased, ultimately retaining 9–17 non-zero coefficients based on optimal model selection criteria determined through binomial deviance minimization (**Figures 4A, B**). For MASLD analysis, key biomarkers including IGF1, CD38, and ZNF285 emerged as consistently important features across multiple validation runs. Biomarker importance ranking revealed differential contribution values, highlighting the relative significance of each molecular signature in disease classification (**Figures 4C, D**). Parallel LASSO regression analysis of IVDD datasets employed identical methodological approaches, successfully identifying key biomarkers specific to intervertebral disc degeneration pathology (**Figures 4E–H**). Comparative analysis through Venn diagram intersection of MASLD-associated (6 biomarkers) and IVDD-associated (8 biomarkers) signatures revealed four common biomarkers with potential pan-disease relevance, including STAB2, RAPGEFL1, IGF1, and ZNF285 (**Figure 4I**).

### Functional characterization and experimental validation of disease-associated biomarkers

**Figure 5:**
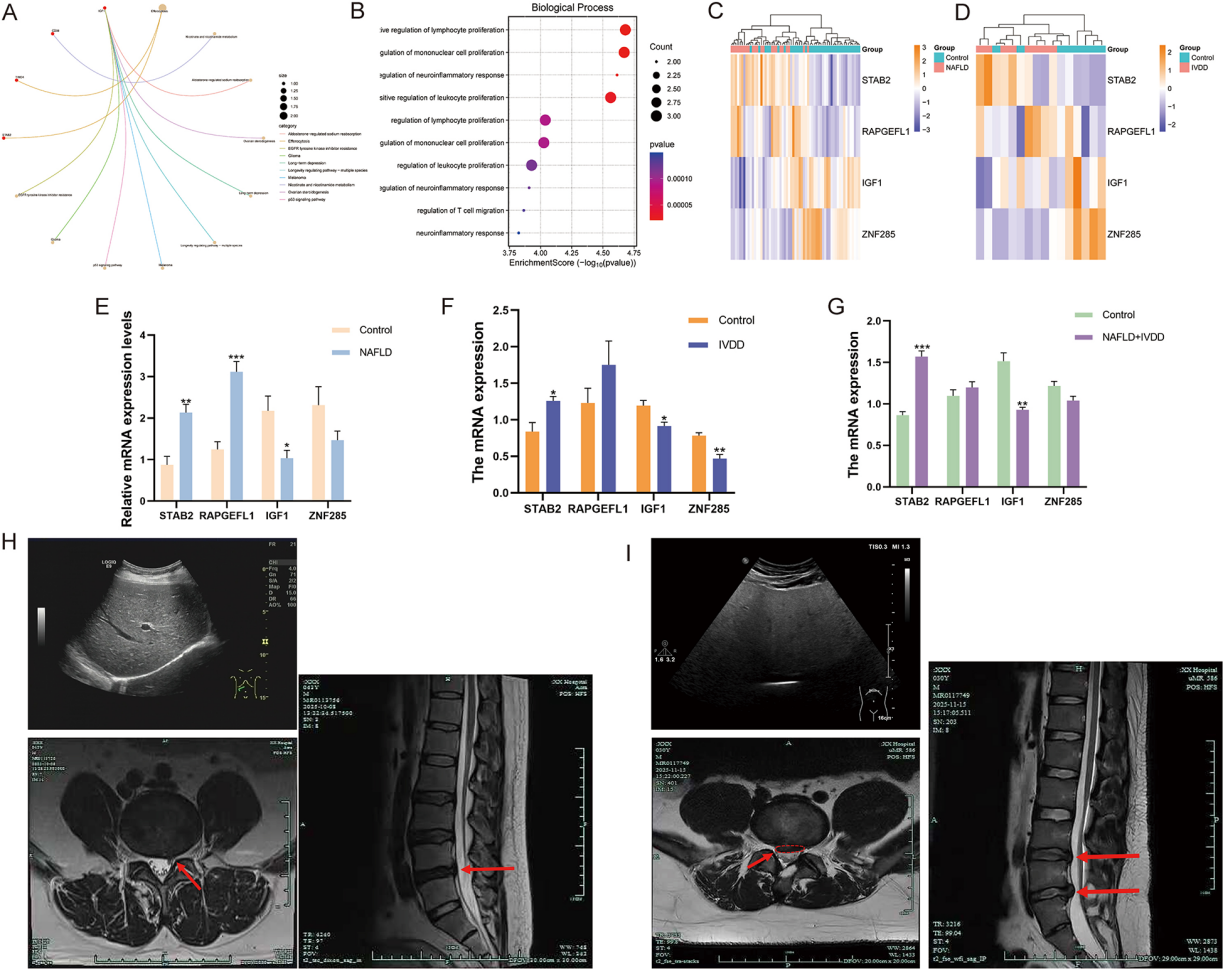
Functional network characterization and experimental validation of key biomarkers. **(A)** Network visualization depicting functional associations between MASLD and IVDD biomarker panels and their enriched biological processes. **(B)** Detailed biomarker-process interaction map illustrating the complex regulatory networks underlying disease pathogenesis. **(C)** Cluster heatmap of four common biomarkers in MASLD. **(D)** Cluster heatmap of four common biomarkers in IVDD. **(E)** The mRNA expression between control and MASLD. **(F)** The mRNA expression between control and IVDD. **(G)** The mRNA expression between control and the group with comorbidities of MASLD-IVDD. **(H)** Representative lumbar spine MRI of isolated IVDD patient. **(I)** Representative lumbar spine MRI of MASLD-IVDD comorbidity patient. Data presented as mean ± SEM. *p<0.05, **p<0.01, ***p<0.001 vs. control group.

Following LASSO regression analysis, we proceeded with comprehensive functional characterization of the identified biomarker panel, comprising 6 MASLD-associated genes and 8 IVDD-associated genes. Network topology analysis and functional annotation revealed three interconnected functional clusters with distinct biological roles: proliferation regulation (IGF1, CD38), inflammatory control (STAB2, CD200R1), and metabolic-immune interface (ZNF285, RAPGEFL1). Notably, IGF1 emerged as a critical hub node bridging metabolic and immune pathways, highlighting its central role in disease pathogenesis (**Figures 5A, B**). Venn diagram intersection analysis identified four shared biomarkers between MASLD and IVDD: STAB2, RAPGEFL1, IGF1, and ZNF285, which demonstrated consistent expression patterns across both diseases. Clustering heatmap analysis revealed that STAB2 and RAPGEFL1 exhibited elevated expression in both MASLD and IVDD conditions, while IGF1 and ZNF285 showed decreased expression patterns in both diseases (**Figures 5C, D**). To validate these bioinformatics findings, quantitative RT-PCR analysis was performed using peripheral blood samples from patients and healthy controls. The selection of these four biomarkers for experimental validation was based on multiple convergent criteria: they represented the complete intersection of MASLD-associated (6 biomarkers) and IVDD-associated (8 biomarkers) LASSO-identified signatures, suggesting potential pan-disease relevance; they demonstrated robust coefficient values in both disease models (|coefficient| > 0.3); and preliminary literature review indicated their involvement in metabolic and inflammatory pathways relevant to both conditions. Furthermore, all four biomarkers showed detectable expression in peripheral blood transcriptomes, making them practical candidates for clinical validation. In MASLD patients compared to healthy controls, we observed significant upregulation of STAB2 and RAPGEFL1 expression (p<0.01, p<0.001), accompanied by notable downregulation of IGF1 (p<0.05). Similarly, IVDD patients demonstrated elevated STAB2 expression alongside decreased IGF1 and ZNF285 expression levels (p<0.01, p<0.05). Particularly intriguing was our analysis of patients presenting with concurrent MASLD-IVDD comorbidity, which revealed significant STAB2 upregulation and pronounced IGF1 downregulation (p<0.01), suggesting these biomarkers may serve as indicators of disease severity or multi-system involvement.

Clinical imaging validation further corroborated the molecular findings in patients with IVDD and concurrent MASLD-IVDD. Magnetic resonance imaging (MRI) examination of IVDD patient revealed characteristic degenerative changes including straightening of lumbar lordosis, marginal osteophyte formation at vertebral bodies, sacralization of S1 vertebra, and slight concavity at L2, L4, and L5 endplates (**Figure 5H**). Notably, a small region of increased signal intensity on FS-T2WI was observed at the L4 endplate. The L4/5 and L5/S1 intervertebral discs demonstrated reduced signal intensity with bulging toward the periphery, resulting in compression of the dural sac and mild bilateral foraminal stenosis. The spinal cord, conus medullaris, filum terminale, and cauda equina exhibited normal morphology and signal characteristics. Lumbar paraspinal soft tissues showed mild edema. The radiological diagnosis confirmed L4/5 and L5/S1 intervertebral disc degeneration with disc bulging, lumbar degenerative changes, Schmorl’s nodes at L2, L4, and L5 vertebral bodies, and suspected L4 endplate inflammation (Modic changes). In contrast, MRI examination of patient with concurrent MASLD-IVDD comorbidity demonstrated more pronounced degenerative features despite similar disc involvement (**Figure 5I**). Imaging revealed straightening of lumbar lordosis with mild localized superior endplate concavity at L2-L4 vertebrae. The L4–5 and L5-S1 intervertebral discs showed markedly reduced signal intensity with focal posterior herniation, causing dural sac compression and left-sided L4–5 foraminal narrowing. Mild dilation with fluid accumulation was observed in the left S2–3 sacral foramen. The radiological diagnosis included L4–5 and L5-S1 intervertebral disc degeneration with herniation, mild spinal canal and left L4–5 foraminal stenosis, straightening of lumbar curvature, suspected Schmorl’s nodes at L2-L4 superior endplates, and mild dilation with fluid in the left S2–3 sacral foramen. Importantly, hepatic imaging in this patient showed normal liver parenchyma signal characteristics on MRI, consistent with early-stage MASLD without advanced structural changes, suggesting that metabolic dysfunction can influence disc degeneration even in the absence of severe hepatic structural alterations. The comparison between isolated IVDD (**Figure 5H**) and MASLD-IVDD comorbidity (**Figure 5I**) patients revealed qualitatively similar patterns of disc degeneration (reduced disc signal, posterior bulging/herniation, foraminal stenosis), supporting the notion that both conditions share common degenerative pathways. However, the MASLD-IVDD patient exhibited more extensive endplate changes (L2-L4 involvement versus L2, L4, L5 in isolated IVDD) and additional features such as sacral foramen changes, potentially reflecting more systemic involvement. These imaging findings provide clinical validation for our molecular observations that MASLD and IVDD share pathogenic mechanisms, and that their combination may reflect enhanced systemic metabolic and inflammatory perturbations affecting spinal structures.

### Single-cell atlas construction and disease-associated cell population identification

**Figure 6:**
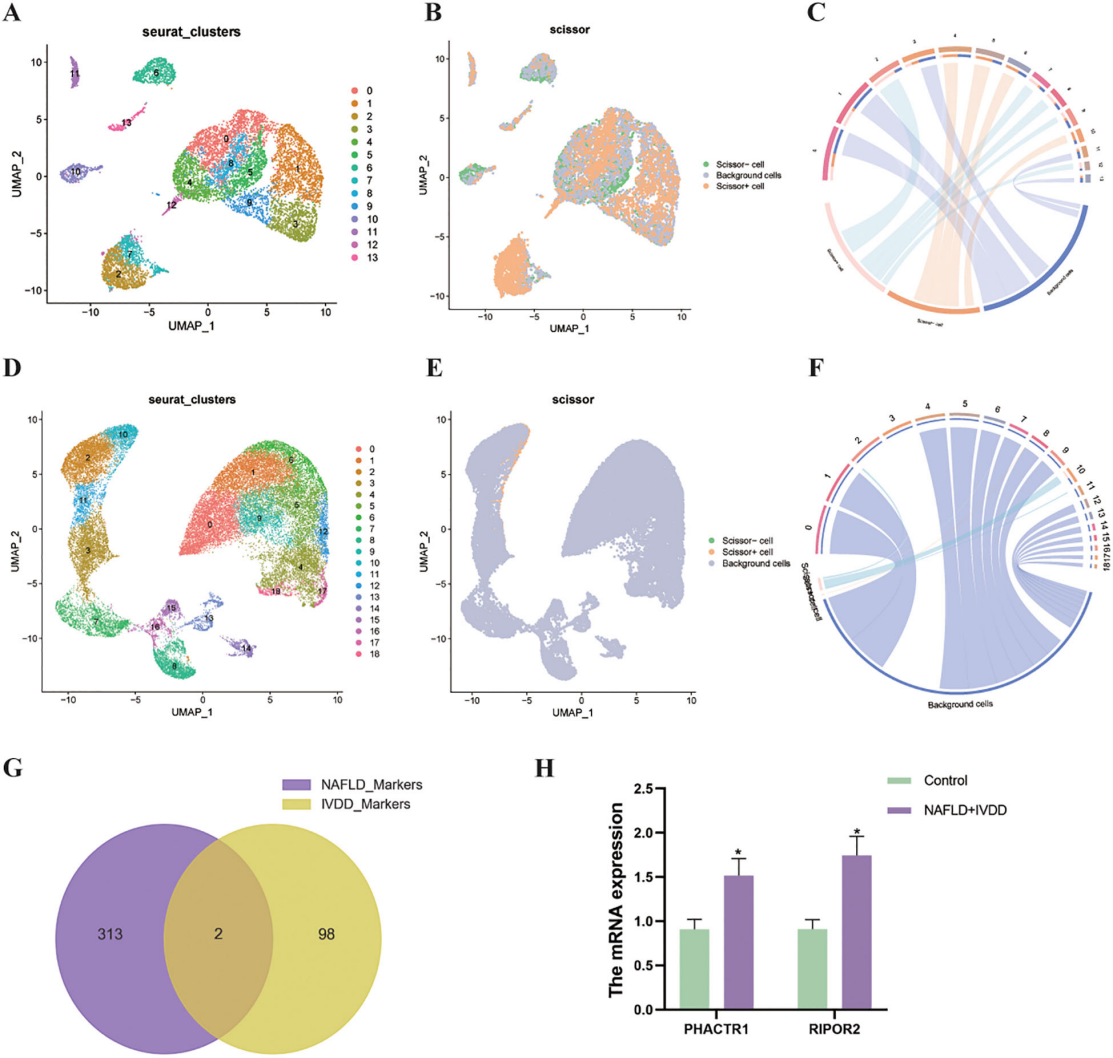
Single-cell characterization of disease-associated cellular populations through integrative Scissor analysis. **(A, B)** MASLD single-cell landscape: **(A)** Unsupervised UMAP clustering of all cells; **(B)** Disease-associated subsets identified by Scissor (Scissor+ cells represent MASLD-positively correlated populations, Scissor- cells represent MASLD-negatively correlated populations). **(C)** Chord plot visualization showing relationships between Scissor-identified cell subsets and associated phenotypes. **(D, E)** IVDD single-cell characterization: **(D)** Comprehensive UMAP clustering; **(E)** Scissor-identified disease-correlated cellular subsets (Scissor+ cells represent IVDD-positively correlated populations, Scissor- cells represent IVDD-negatively correlated populations). **(F)** Phenotype-cell subset relationship analysis through chord plot visualization. **(G)** Venn diagram intersection identifying common highly variable genes in disease-positively correlated cells across both conditions. **(H)** The highly variable genes expression between control and the group with comorbidities of MASLD-IVDD. Data presented as mean ± SEM. *p<0.05 vs. control group.

High-quality single-cell transcriptomic datasets were established following rigorous quality control procedures monitoring mitochondrial gene expression and blood cell contamination. After comprehensive filtering, 10,388 MASLD-derived cells and 35,846 IVDD-derived cells met quality standards for downstream analysis. Uniform Manifold Approximation and Projection (UMAP) dimensionality reduction successfully partitioned MASLD tissue cells into 14 distinct clusters (**Figure 6A**) and IVDD tissue cells into 19 clusters (**Figure 6D**), revealing the cellular heterogeneity underlying disease pathology. Integration of single-cell and bulk RNA-seq datasets through Scissor analysis enabled identification of disease-correlated cell populations. Binary phenotype classification (disease samples = 1, healthy controls = 0) facilitated the detection of Scissor+ cells (disease-positively correlated) and Scissor- cells (disease-negatively correlated) within each dataset (**Figures 6B, E**). Hypergeometric distribution analysis validated the statistical significance of cell subset enrichment patterns, with results visualized through chord plots demonstrating phenotype-cell subset relationships (**Figures 6C, D**). Analysis revealed that cluster 2 in MASLD data corresponded to disease-associated cell subpopulations, while cluster 10 in IVDD data represented the primary disease-correlated cellular subset. Highly variable gene identification within disease-associated cell subpopulations using the “FindAllMarkers” function (LogFC threshold = 0.25, min.pct = 0.1) followed by Venn diagram intersection analysis yielded two common biomarkers (PHACTR1 and RIPOR2) for subsequent experimental validation (**Figure 6G**). Further verification revealed that in patients with comorbid MASLD-IVDD, the expression of the PHACTR1 and RIPOR2 genes were increased (p< 0.05) (**Figure 6H**).

### Cellular diversity and biomarker localization in disease microenvironments

**Figure 7:**
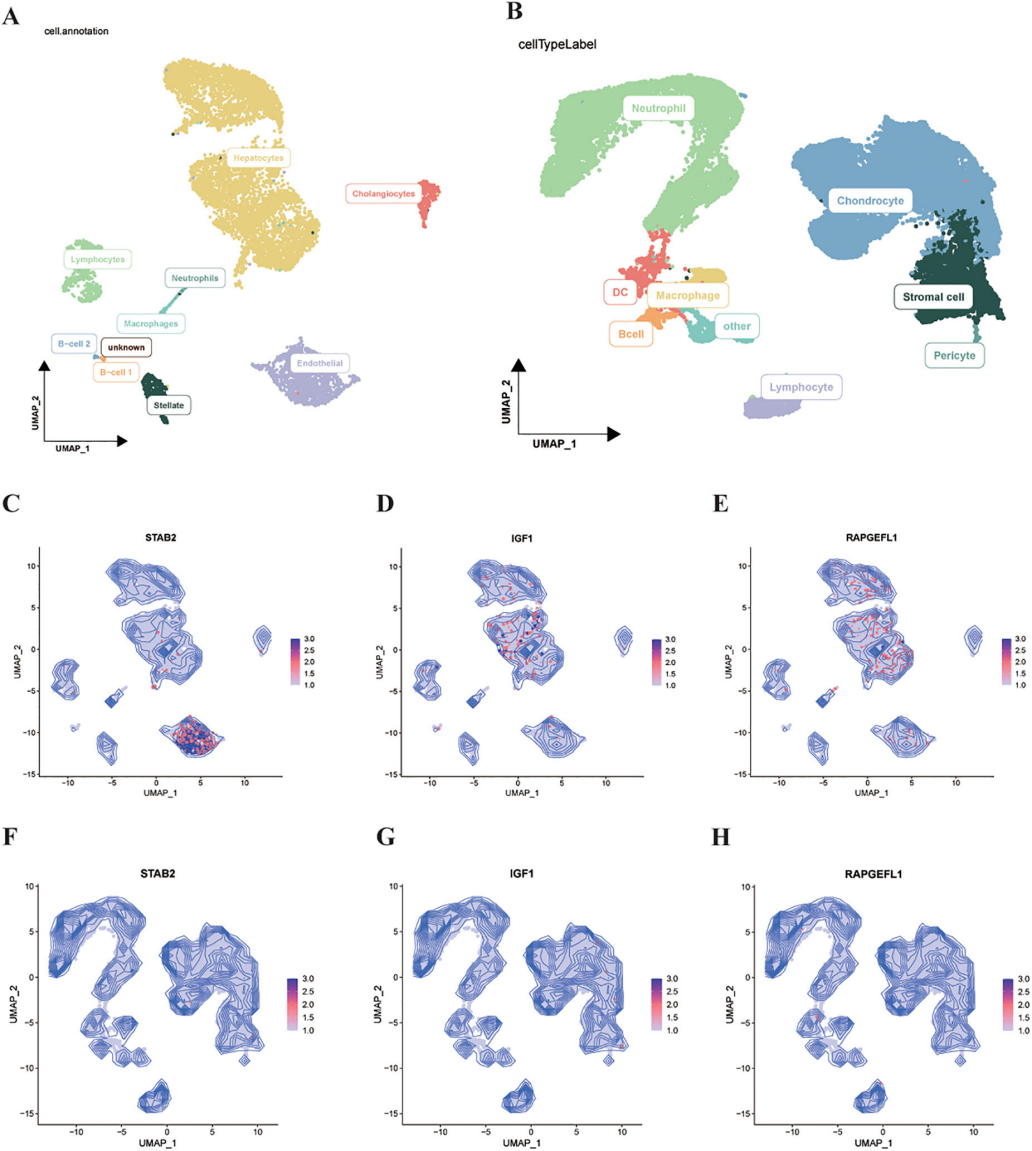
Comprehensive cellular landscape and biomarker distribution analysis in disease tissues. **(A)** UMAP projection of MASLD cellular populations identified through single-cell RNA sequencing, with color-coded annotation indicating distinct cell types. **(B)** IVDD cellular population mapping showing annotated cell type diversity. **(C–E)** Contour density visualization demonstrating STAB2, IGF1, RAPGEFL1 enrichment and localization patterns within GSE202379 single-cell data. **(F–H)** Contour density visualization demonstrating STAB2, IGF1, RAPGEFL1 enrichment and localization patterns within GSE153066 single-cell data.

Cell type annotation based on established marker genes and database references resulted in the identification of 9 distinct cell populations in MASLD tissues and 8 cell types in IVDD tissues (**Figures 7A, B**). This comprehensive cellular atlas provided insights into the diverse microenvironmental compositions underlying disease pathology. Contour density mapping was employed to examine the spatial distribution and enrichment patterns of the four key biomarkers identified through bulk RNA-seq analysis. Interestingly, while these biomarkers demonstrated varying degrees of enrichment and localization within MASLD cellular populations, their expression patterns were less pronounced in IVDD tissue data (**Figures 7C–H**). This observation suggests potential disease-specific biomarker expression profiles, possibly influenced by differences in sample sizes or disease-specific cellular compositions. Biomarker localization analysis revealed cell-type-specific expression patterns. In MASLD tissues, STAB2 was predominantly enriched in endothelial cells and hepatocytes. IGF1 showed broader expression across hepatocytes and hepatic stellate cells, reflecting its production by these cell types. In IVDD tissues, while the four bulk RNA-seq-derived biomarkers (STAB2, RAPGEFL1, IGF1, ZNF285) showed relatively lower expression intensity compared to MASLD tissues—possibly reflecting differences in sample size or disease-specific cellular compositions—detectable expression was observed across multiple cell types including chondrocyte cells, stromal cell, and immune cell populations, suggesting their involvement in disc tissue pathophysiology (**Figures 7C–H**).

### Immune microenvironment communication networks in MASLD and IVDD pathology

**Figure 8:**
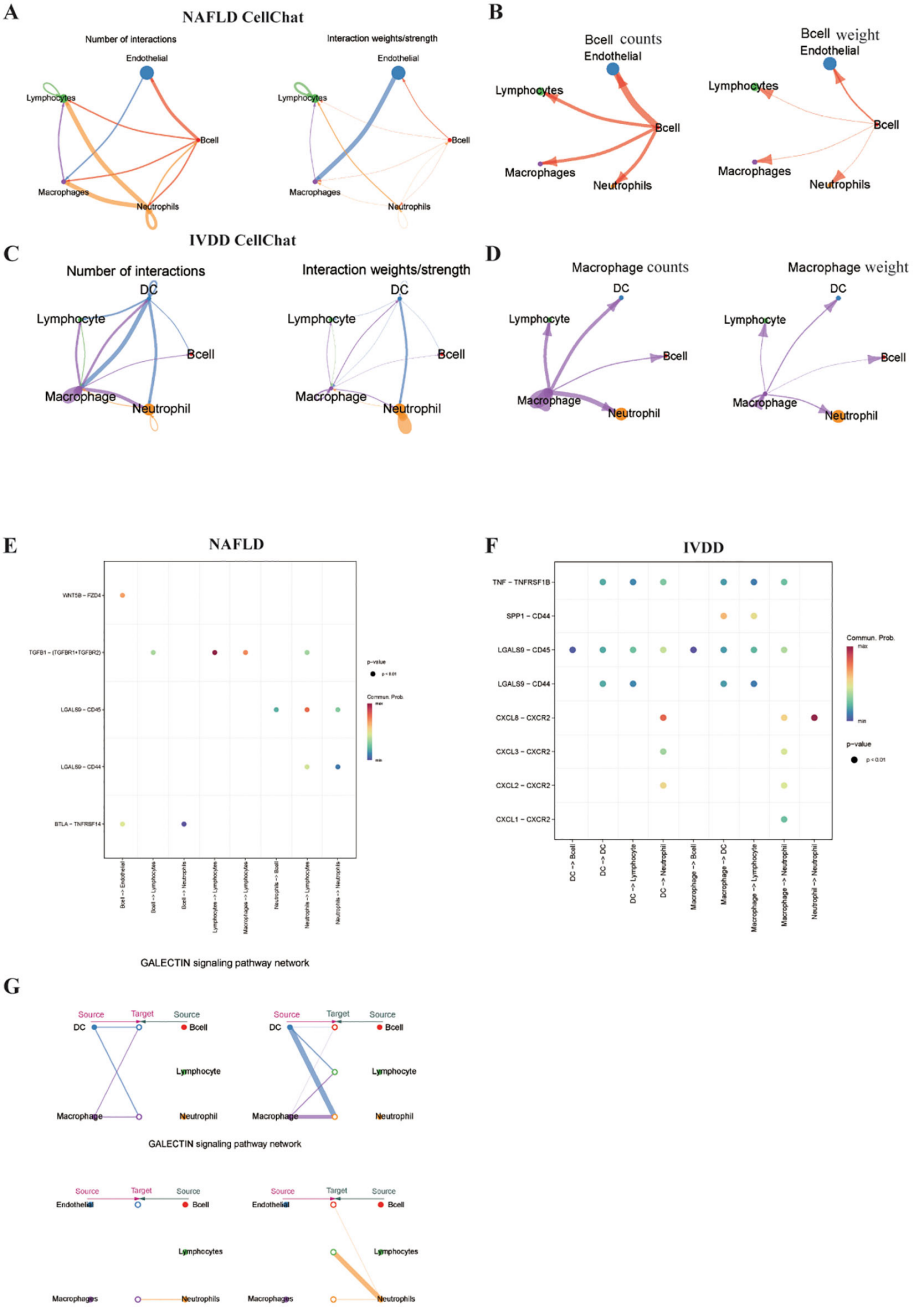
Immune cell interaction networks and communication pathway analysis in MASLD and IVDD. **(A–D)** Intercellular communication analysis identifying cell types with highest communication status and intensity within MASLD and IVDD immune microenvironments. **(E, F)** Receptor-ligand enrichment bubble charts displaying primary interaction pairs in MASLD and IVDD datasets. **(G)** Comparative analysis revealing GALECTIN as the predominant shared signaling pathway in immune cell communications between both diseases.

Focused analysis of immune cell populations extracted from both datasets revealed distinct intercellular communication patterns using CellChat package analysis (**Figures 8A–D**). Our findings demonstrate that B cells serve as dominant communicators in MASLD immune interactions, while macrophages emerge as central regulators in IVDD immune networks. Comparative pathway analysis identified GALECTIN signaling as the predominant shared communication pathway between immune cells in both diseases (**Figure 8G**), suggesting common immunological mechanisms despite different primary pathologies. Receptor-ligand enrichment analysis revealed limited overlap in significantly enriched pathways between the two diseases (**Figures 8E, F**), potentially reflecting the distinct immunological landscapes or influenced by the relatively modest immune cell representation in the single-cell datasets. This observation highlights the need for expanded immune cell profiling to fully characterize disease-specific communication networks.

## Discussion

This study conducted a comprehensive bioinformatics investigation of the molecular crosstalk between MASLD and IVDD. Through integrated analysis of bulk RNA sequencing and single-cell RNA sequencing datasets, we revealed common pathogenic mechanisms and potential therapeutic targets, furthering our understanding of how systemic metabolic dysfunction influences distant tissue pathological processes.

Transcriptomic profiling analysis revealed distinct yet interconnected molecular signatures, with MASLD demonstrating pronounced enrichment in metabolic regulatory pathways including cholesterol metabolism, glutathione metabolism, and PPARγ signaling cascades, while IVDD exhibited significant activation of cellular signaling networks predominantly featuring MAPK cascades, PI3K-AKT pathway, and HIF-1 signaling. This dichotomy suggests that MASLD represents the source of systemic metabolic dysfunction, whereas IVDD manifests as the downstream consequence of altered metabolic homeostasis in glucose-dependent tissues (34, 35). The enrichment of cholesterol and lipid metabolism pathways in MASLD directly supports the notion that disrupted lipid homeostasis serves as a primary driver of hepatic steatosis, while the activation of hypoxia-responsive HIF-1 signaling in IVDD demonstrates that disc cells, being highly sensitive to nutrient availability and pH balance, respond to systemic metabolic perturbations through adaptive stress response mechanisms (36, 37). Through machine learning approaches, four common biomarkers—STAB2, RAPGEFL1, IGF1, and ZNF285—were identified, and subsequent validation using blood samples revealed that STAB2 and IGF1 retained statistical significance in patients with concurrent MASLD and IVDD comorbidities.

STAB2 (Stabilin-2), a multifunctional scavenger receptor belonging to the hyaluronan receptor for endocytosis (HARE) family (38), showed apparent upregulation in both diseases, which may reflect its role in responding to metabolic stress and oxidative damage. STAB2 is predominantly expressed in liver sinusoidal endothelial cells and tissue macrophages, where it functions as a clearance receptor for modified lipoproteins, oxidized low-density lipoproteins, and apoptotic cells (38–41). In the context of MASLD, elevated STAB2 expression appears consistent with the liver’s adaptive response to increased oxidative stress and lipid peroxidation that characterize hepatic steatosis. MASLD is associated with enhanced reactive oxygen species production and compromised antioxidant defense systems, leading to lipid oxidation and cellular damage (42). The upregulation of STAB2 may represent a compensatory mechanism attempting to clear these oxidatively modified lipoproteins and damaged cellular components that accumulate during the progression from simple steatosis to more severe hepatic dysfunction. This clearance function becomes particularly important as MASLD progresses and oxidative stress intensifies, potentially contributing to the chronic low-grade inflammatory state that may develop (43). In IVDD, the upregulation of STAB2 may indicate enhanced tissue remodeling and debris clearance within the degenerating disc microenvironment, where oxidative stress and cellular senescence lead to accumulation of damaged extracellular matrix components. Therefore, systemic metabolic dysfunction can affect the pathological changes in distant tissues.

IGF1, a 70-amino acid polypeptide hormone structurally similar to insulin, serves as a master regulator of cellular growth, survival, differentiation, and metabolic homeostasis through its binding to the IGF1 receptor and subsequent activation of multiple downstream signaling cascades including the PI3K-AKT and MAPK pathways (44). In hepatic tissue, IGF1 is primarily synthesized by hepatocytes under the regulation of growth hormone and nutritional status, playing crucial roles in glucose homeostasis, lipid metabolism, and hepatocyte regeneration (45–47). The observed downregulation of IGF1 in MASLD may reflect the hepatocyte dysfunction and impaired synthetic capacity that result from the complex metabolic dysregulation characterizing this condition, including lipotoxicity, oxidative stress, and altered cellular energy production. This creates a systemic consequence where reduced hepatic IGF1 production compromises metabolic homeostasis and tissue repair mechanisms throughout the body. In the context of IVDD pathology, this systemic IGF1 deficiency may further demonstrate that systemic metabolic dysfunction can affect glucose-dependent tissues. Disc cells rely heavily on glucose metabolism and are sensitive to changes in nutrient availability, pH balance, and oxidative stress (48). IGF1 plays particularly critical roles in maintaining disc cell viability and promoting extracellular matrix synthesis (49, 50). The decreased systemic IGF1 levels associated with MASLD could directly compromise disc cell metabolic activity, reduce collagen and proteoglycan synthesis, and impair the disc’s capacity for self-repair and regeneration, thereby providing a direct molecular mechanism linking hepatic metabolic dysfunction to disc degeneration (51, 52). The insulin-like metabolic effects of IGF1 further suggest that its downregulation could contribute to local insulin resistance within disc tissues, directly supporting our proposition that MASLD-associated insulin resistance and glucose metabolic abnormalities create systemic metabolic perturbations that affect glucose-dependent tissues like the intervertebral disc. To provide mechanistic context for our identified biomarkers, we performed comprehensive literature mining and pathway analysis. STAB2 has been extensively characterized as a scavenger receptor responsible for clearing oxidized lipoproteins, advanced glycation end products, and cellular debris (40), functions that are directly relevant to both MASLD-associated oxidative stress and disc degeneration-related tissue remodeling. In particular, STAB2’s role in binding and endocytosing oxidized low-density lipoproteins (oxLDL) in liver sinusoidal endothelial cells has been well-documented (39, 53), and recent studies have shown that STAB2 levels are significantly elevated in individuals with higher atherosclerotic plaque burden and correlate with dysregulated lipid metabolism (54). Similarly, IGF1’s roles in glucose metabolism, PI3K-AKT and MAPK signaling activation, and extracellular matrix synthesis have been well-documented across multiple tissue types (44). Specifically for intervertebral disc biology, IGF1 has been shown to stimulate proteoglycan synthesis, promote nucleus pulposus cell proliferation, and induce extracellular matrix production via the PI3K-AKT and ERK/MAPK signaling pathways (55, 56).

The single-cell analysis results provide initial insights into the cellular heterogeneity underlying both diseases and suggest how metabolic dysfunction might manifest at the cellular level. The identification of disease-associated cell populations through Scissor analysis and the discovery of potentially common highly variable genes (PHACTR1 and RIPOR2) in these populations suggests that similar cellular stress responses and adaptive mechanisms may be activated in both conditions. PHACTR1 (Phosphatase and Actin Regulator 1) plays roles in cellular cytoskeletal organization and has been implicated in metabolic regulation, while RIPOR2 (RHO Family Interacting Cell Polarization Regulator 2) is involved in cellular polarity and migration (57–59). The apparent upregulation of both genes in patients with comorbid MASLD and IVDD may indicate enhanced cellular remodeling and stress response programs, which could represent compensatory mechanisms attempting to maintain tissue homeostasis under metabolic stress. The varying biomarker enrichment patterns between MASLD and IVDD cellular populations suggest tissue-specific responses to common systemic signals, which may explain why metabolic dysfunction manifests differently across organ systems while maintaining underlying mechanistic similarities. Our immune microenvironment analysis reveals another layer of complexity in MASLD-IVDD crosstalk, with B cells dominating communication networks in MASLD while macrophages serve as central regulators in IVDD immune interactions. The identification of GALECTIN signaling as the predominant shared communication pathway provides mechanistic insight into how immune dysregulation contributes to both diseases. Galectins are β-galactoside-binding lectins that regulate immune cell activation, apoptosis, and tissue remodeling, and their involvement in both diseases suggests that common immunoregulatory mechanisms operate across different tissue environments (60). This finding appears to support the concept that metabolic dysfunction could promote a systemic inflammatory state that might influence immune cell behavior in distant tissues.

Our identification of shared molecular signatures between MASLD and IVDD carries important clinical implications. The blood-based biomarkers STAB2 and IGF1, which showed consistent alterations across both diseases with enhanced changes in comorbid patients, could enable early detection of IVDD in MASLD patients, risk stratification for multi-system involvement, and minimally invasive disease monitoring. Therapeutically, our findings suggest that interventions targeting systemic metabolic dysfunction could provide dual benefits: IGF1-targeted therapies could theoretically improve both hepatic metabolic function and disc cell viability given IGF1’s documented roles in glucose metabolism and extracellular matrix synthesis, while anti-inflammatory approaches targeting shared pathways like GALECTIN signaling could address chronic inflammation across both tissues. More broadly, comprehensive metabolic management may prevent both MASLD and IVDD progression, supporting integrated care models. However, clinical translation requires larger validation cohorts to establish diagnostic performance, longitudinal studies to determine temporal relationships and prognostic value, and cost-effectiveness analyses. Despite these limitations, our findings provide a foundation for developing integrated diagnostic and therapeutic strategies for metabolic diseases with multi-organ manifestations, addressing a critical need given the rising prevalence of both MASLD and IVDD in aging populations.

The limitations of our study should be acknowledged. The reliance on publicly available datasets may introduce batch effects and heterogeneity in sample preparation and analysis protocols, although we employed rigorous normalization and batch correction procedures to minimize these effects. The relatively modest representation of immune cells in the single-cell datasets may have limited our ability to fully characterize immune communication networks, highlighting the need for future studies with enhanced immune cell profiling. Additionally, while our experimental validation in human blood samples supports the clinical relevance of our findings, larger validation cohorts and longitudinal studies will be necessary to establish the prognostic and diagnostic utility of these biomarkers.

**Figure 9:**
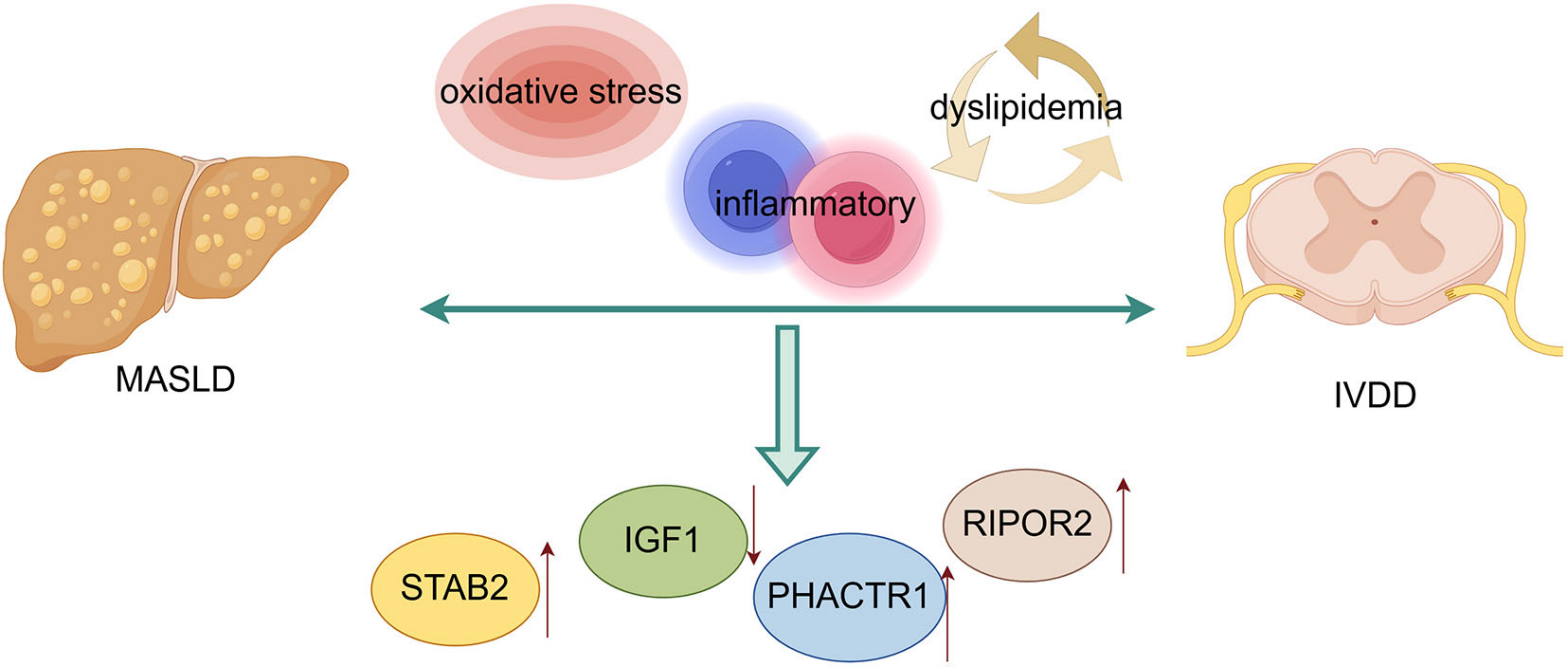
Proposed molecular mechanisms underlying MASLD-IVDD crosstalk.

In conclusion, this study provides compelling evidence for significant molecular crosstalk between MASLD and IVDD, mediated by shared inflammatory, metabolic, and cellular stress response pathways (**Figure 9**). The identification of common biomarkers and molecular mechanisms not only advances our understanding of how systemic metabolic dysfunction influences distant tissue pathology but also opens new avenues for integrated diagnostic and therapeutic approaches.

## Data Availability

The original contributions presented in the study are included in the article/**Supplementary Material**. Further inquiries can be directed to the corresponding author.
