# Supplementary material for: Molecular crosstalk between MASLD and IVDD revealed through integrated biomarker discovery analysis
Source: Front Immunol. 2026 Jan 26;17:1703972. doi: 10.3389/fimmu.2026.1703972 (PMC12883656; doi:10.3389/fimmu.2026.1703972)
Supplement: Supplementary file 1 [file Table1.docx]

Supporting Information for

**Molecular Crosstalk Between NAFLD and IVDD Revealed Through Integrated Biomarker Discovery Analysis**

**Table S1. Bulk RNA-seq datasets of NAFLD and IVDD.**

| **Datasets** | **Samples** | **Download** | **Disease** |
| --- | --- | --- | --- |
| GSE61260 | 61 | [GEO](https://www.ncbi.nlm.nih.gov/geo/) | NAFLD |
| GSE124272 | 16 | [GEO](https://www.ncbi.nlm.nih.gov/geo/) | IVDD |

Table S2. Single-cell RNA-seq datasets of NAFLD and IVDD.

| **Datasets** | **Patients** | **samples** | **Download** | **Disease** |
| --- | --- | --- | --- | --- |
| GSE202379 | 3 | 10388 | [GEO](https://www.ncbi.nlm.nih.gov/geo/) | NAFLD |
| GSE153066 | 8 | 35846 | [GEO](https://www.ncbi.nlm.nih.gov/geo/) | IVDD |

Table S3. The primers for the target genes.

| Gene | Forward primer | Reverse primer |
| --- | --- | --- |
| STAB2 | AGGTGGGCTATGTGGGAGAT | CGCCCAGACAAGGTCTCATT |
| RAPGEFL1 | GTGGCAATGCTAATTCACCCA | AATGTCATCCAGCAGGATATAGAAA |
| IGF1 | TGCTCTCAACATCTCCCATCTC | ACCCTGTGGGCTTGTTGAAA |
| ZNF285 | CCTCATGTTAGTGAGAGACGGG | AGAGAAATGCCTGCCCACTC |
| PHACTR1 | TCCGTTGCTAAGGTGTTCCA | CTGCCCCCAGGTTAAAGGAG |
| RIPOR2 | CGCCTGTCCTCTCCAAGATG | TGGTCGGTAGTCCTTCACCA |

**Supporting Information Figure**


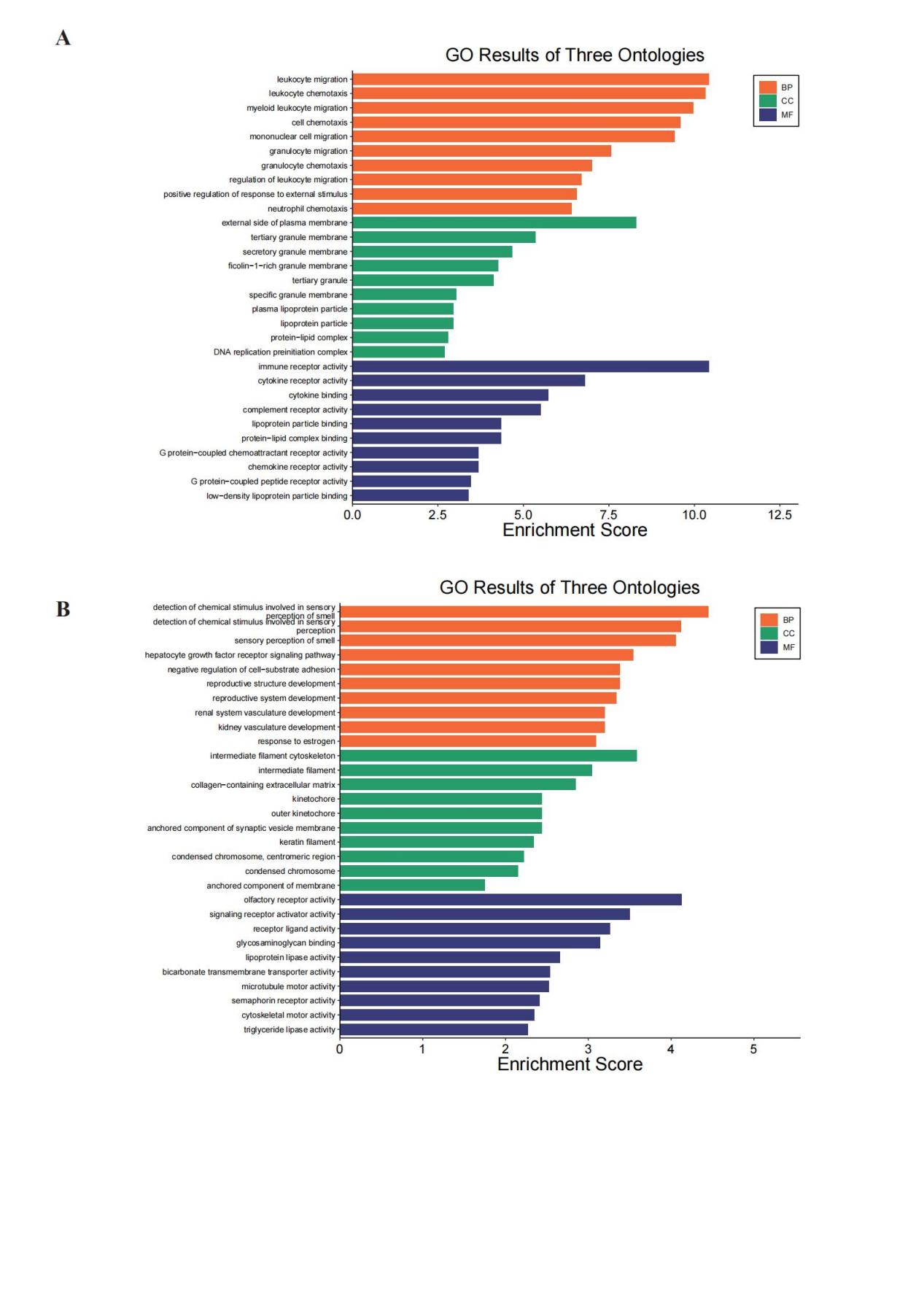


**Figure S1.** (A) Enrichment analysis of GO pathways for screening key genes in NAFLD disease. (B) Enrichment analysis of GO pathways for screening key genes in IVDD disease.


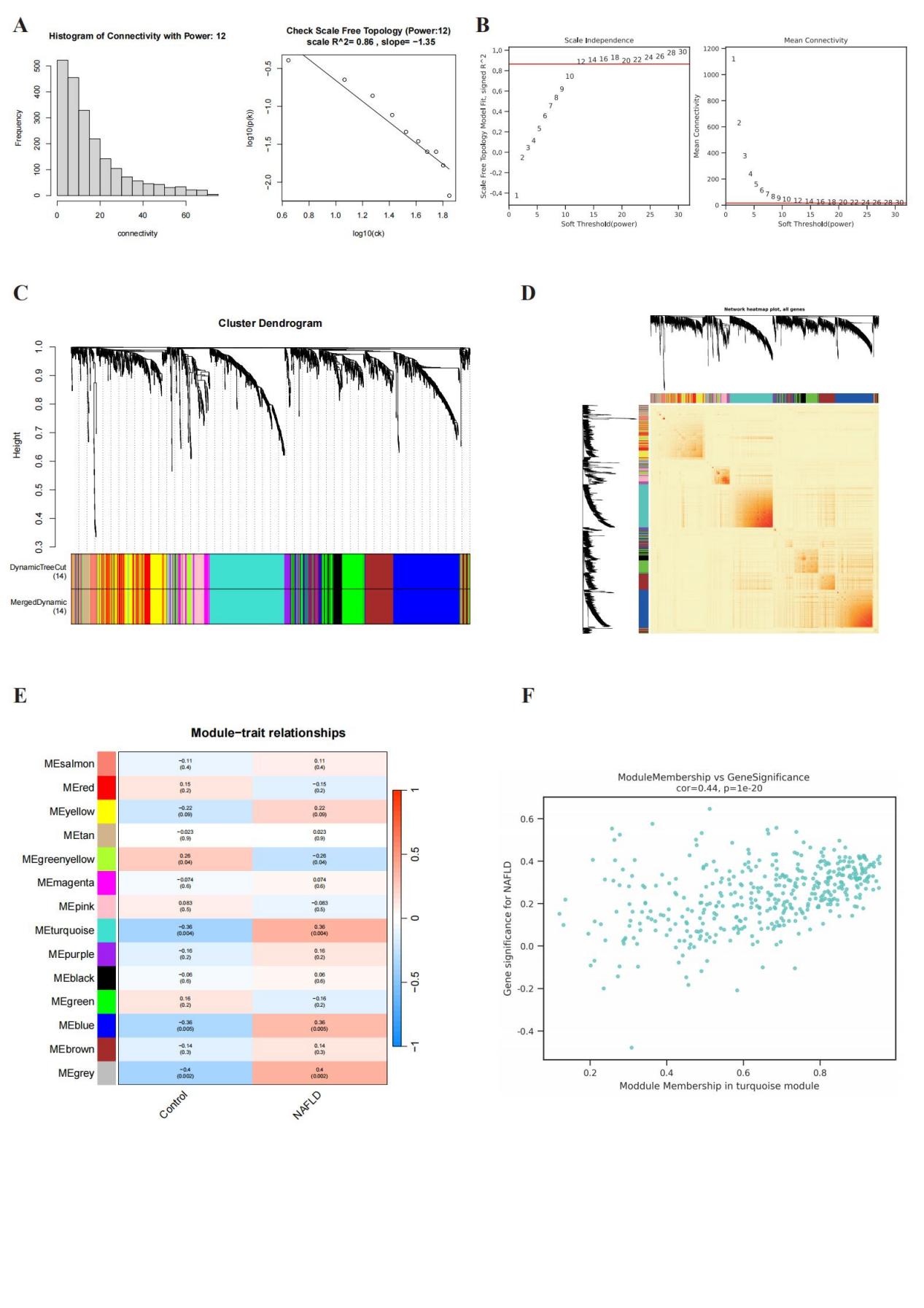


**Figure S2.** Hierarchical clustering analysis of gene co-expression patterns in NAFLD progression. (A-B) Dendrograms showing hierarchical clustering of genes based on topological overlap matrix (TOM) dissimilarity, using average linkage method. The y-axis represents branch height, indicating the degree of similarity between gene expression profiles. (C) Initial gene clustering using DynamicTreeCut algorithm, identifying 14 preliminary gene modules. (D) Final merged clustering resulting in 14 biologically meaningful co-expression modules after dynamic branch cutting. (E-F) Detailed views of selected branches from the dendrogram, highlighting: Distinct module separation (colored branches), Module-specific gene expression patterns, Key functional annotations for representative modules.


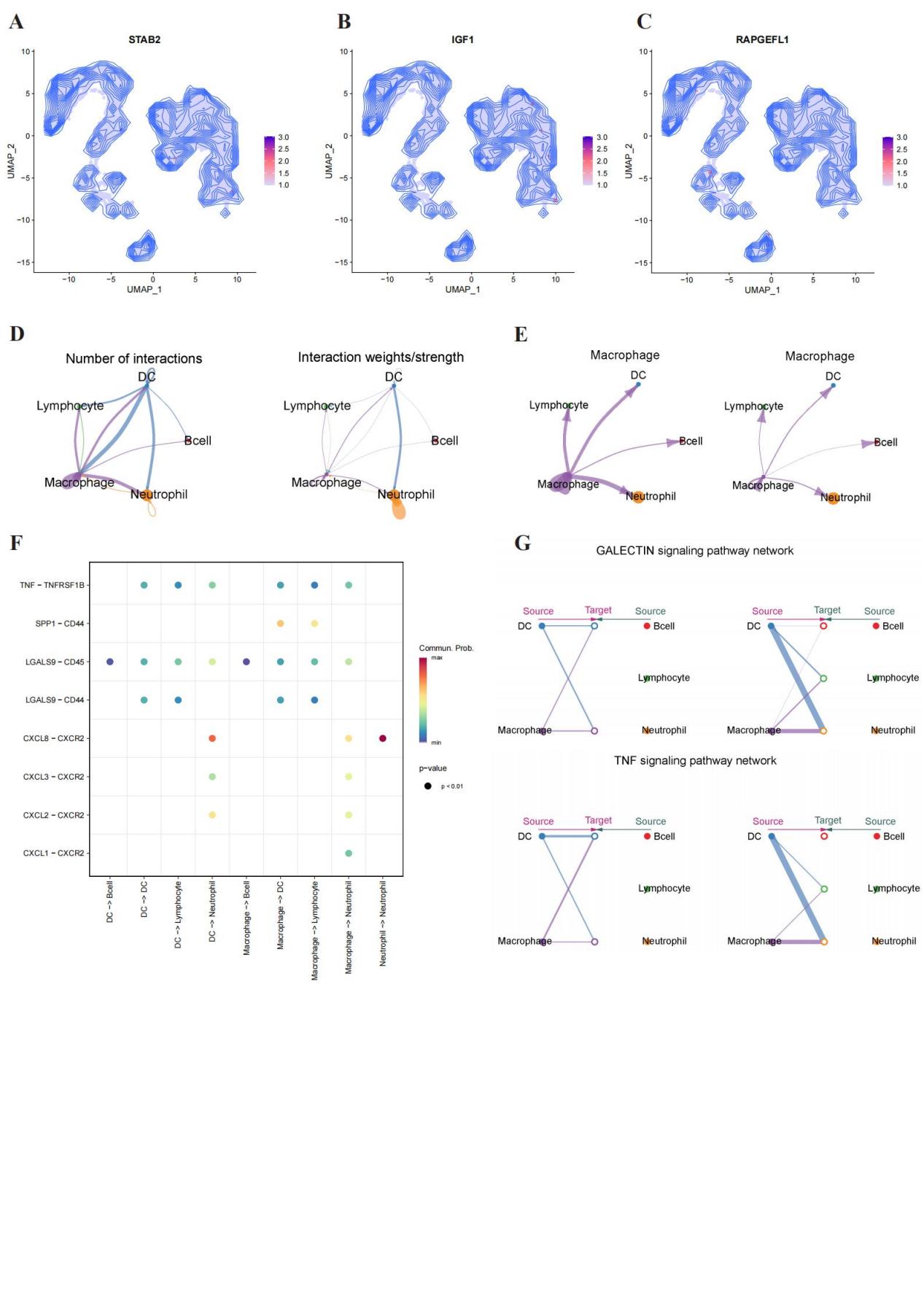


**Figure S3.** (A-C) The contour density map shows the enrichment and localization of key markers in single-cell data. (D-E) Intercellular communication analysis demonstrated the communication status of immune cells in IVDD and the cell types with the highest communication intensity. (F) The enrichment bubble chart shows the main receptor-ligand pairs in the IVDD dataset. (G) The results of intercellular communication analysis show that the common signaling pathway of immune cells in these two diseases is mainly GALECTIN.
